# Pepsin Digest of Wheat Gliadin Fraction Increases Production of IL-1β via TLR4/MyD88/TRIF/MAPK/NF-κB Signaling Pathway and an NLRP3 Inflammasome Activation

**DOI:** 10.1371/journal.pone.0062426

**Published:** 2013-04-29

**Authors:** Lenka Palová-Jelínková, Klára Dáňová, Hana Drašarová, Miloš Dvořák, David P. Funda, Petra Fundová, Anna Kotrbová-Kozak, Marie Černá, Jana Kamanová, Stefan F. Martin, Marina Freudenberg, Ludmila Tučková

**Affiliations:** 1 Institute of Microbiology, Department of Immunology, Academy of Sciences of the Czech Republic, Prague, Czech Republic; 2 1^st^ Medical Faculty, Charles University, Prague, Czech Republic; 3 Third Faculty of Medicine, Charles University, Prague, Czech Republic; 4 Allergy Research Group, Department of Dermatology, University Medical Center Freiburg, Freiburg, Germany; 5 Max Planck Institute for Immunobiology and Epigenetics, Freiburg, Germany; Instutite of Agrochemistry and Food Technology, Spain

## Abstract

Celiac disease (CD) is a gluten-responsive, chronic inflammatory enteropathy. IL-1 cytokine family members IL-1β and IL-18 have been associated with the inflammatory conditions in CD patients. However, the mechanisms of IL-1 molecule activation in CD have not yet been elucidated. We show in this study that peripheral blood mononuclear cells (PBMC) and monocytes from celiac patients responded to pepsin digest of wheat gliadin fraction (PDWGF) by a robust secretion of IL-1β and IL-1α and a slightly elevated production of IL-18. The analysis of the upstream mechanisms underlying PDWGF-induced IL-1β production in celiac PBMC show that PDWGF-induced *de novo* pro-IL-1β synthesis, followed by a caspase-1 dependent processing and the secretion of mature IL-1β. This was promoted by K+ efflux and oxidative stress, and was independent of P2X7 receptor signaling. The PDWGF-induced IL-1β release was dependent on Nod-like receptor family containing pyrin domain 3 (NLRP3) and apoptosis-associated speck like protein (ASC) as shown by stimulation of bone marrow derived dendritic cells (BMDC) from NLRP3^−/−^ and ASC^−/−^ knockout mice. Moreover, treatment of human PBMC as well as MyD88^−/−^ and Toll-interleukin-1 receptor domain-containing adaptor-inducing interferon-β (TRIF)^−/−^ BMDC illustrated that prior to the activation of caspase-1, the PDWGF-triggered signal constitutes the activation of the MyD88/TRIF/MAPK/NF-κB pathway. Moreover, our results indicate that the combined action of TLR2 and TLR4 may be required for optimal induction of IL-1β in response to PDWGF. Thus, innate immune pathways, such as TLR2/4/MyD88/TRIF/MAPK/NF-κB and an NLRP3 inflammasome activation are involved in wheat proteins signaling and may play an important role in the pathogenesis of CD.

## Introduction

Celiac disease (CD) is an inflammatory T cell-mediated disorder of the small intestine caused by the gluten fraction of wheat or the homologous proteins from barley and rye, in genetically predisposed individuals. An increased number of intraepithelial lymphocytes and lamina propria cells and their activation, followed by villous atrophy and crypt hyperplasia, characterize CD. Both innate and adaptive immune responses contribute to the onset of mucosal inflammation in CD patients [1]. Fragments of gliadin – a major group of proteins in gluten – cross the epithelium, and are presented by antigen presenting cells to the HLA-DQ2 or HLA-DQ8-restricted CD4+ α/β T lymphocytes present in jejunal mucosa [2]. Recently, other nongluten components of wheat (amylase inhibitors) were reported to contribute to the specific response by stimulation of innate immunity cells [3]. The activated gliadin-specific T lymphocytes produce a spectrum of mediators and cytokines of a Th1 profile, mainly IFN-γ. These mechanisms could participate in intestinal tissue damage by contributing to the proinflammatory environment in the tissue and by activating tissue enzymes, including metalloproteases and tissue transglutaminase [4]. Besides IFN-γ, other cytokines such as IL-1β, IL-6, IL-15, IL-23 and TNF-α produced by innate immune cells contribute to the ongoing inflammation in CD [5]–[7].

IL-1β that belongs to the IL-1 cytokine family together with IL-1α, IL-18, and IL-33, has been associated with the inflammatory conditions in CD patients, and was shown to control the secretion of IL-23 leading to a shift to the Th1/Th17 immune pathway [7]–[9]. Production of IL-1β from inflammatory cells such as monocytes and macrophages requires the following steps: the expression of the pro-IL-1β gene and the synthesis of immature pro-IL-1β protein; the cleavage of pro-IL-1β by active caspase-1 to yield the mature form of IL-1β; and the secretion of mature IL-1β from the cells. The generation of mature IL-1β is tightly controlled by a diverse class of cytosolic protein complexes, known as inflammasomes. Several different inflammasomes have been described, of which NLRP3 and NLRP1 (Nod-like receptor family, containing pyrin domain 3 and 1) inflammasomes have been the most intensively studied. Upon sensing danger signals, the NLRP3 proteins oligomerize and recruit caspase-1 through the adaptor protein apoptosis-associated speck like protein (ASC). Subsequently, caspase-1 undergoes an autocatalytic activation that involves the autoproteolytic processing of the 45-kDa pro-caspase-1 into 20- and 10-kDa subunits. In turn, mature caspase-1 cleaves pro-IL-1β, producing mature IL-1β [10].

In macrophages and dendritic cells (DCs), two temporally separate signals are required to yield the active proinflammatory cytokine. The first signal involves the activation of pattern recognition receptors [e.g. Toll like receptors (TLRs) or Nucleotide Oligomerization Domain (NOD)-like receptors] by pathogen- and danger-associated molecular patterns, which triggers the expression of pro-IL-1β via the NF-κB pathway [11]. Then microbial products [e.g. muramyl dipeptide [12], bacterial and viral RNA [13], and pore-forming toxins [14]] or endogenous signals, such as urate crystals [15] and adenosine triphosphate (ATP) [16] as the potential second signals, activate the NLRP3 inflammasome complex. Although the precise mechanism leading to the activation of NLRP3 remains largely unknown, it is proposed that oxidative stress, lysosomal destabilization with cytosolic cathepsin activity and potassium efflux due to the stimulation of ATP-sensitive potassium channels, or pore formation by bacterial toxins, converge into the activation of NLRP3 [17]. In human monocytes, contrary to the two-step signaling system in macrophages and DCs, differential requirements for the activation of the inflammasome were documented [18]. Caspase-1 is constitutively activated in these cells; therefore, a single stimulation event triggers the expression of pro-IL-1β and mature IL-1β release. The second signal is dispensable, because monocytes release endogenous ATP after stimulation, which in turn activate the inflammasome, and induces IL-1β secretion through the P2X7 receptor. IL-1β production is still dependent on the inflammasome components and modulated by K+ efflux [19], [20].

In celiac patients, downstream products of NLRP3 inflammasome activation (such as IL-1β and IL-18) were shown to affect Th1/Th17 responses [7], [21]. However, the mechanism of IL-1β activation has not yet been elucidated. Here, we analyzed the production of IL-1 cytokine family members in human monocytes and PBMC after stimulation with PDWGF, and investigated the upstream mechanism underlying PDWGF-induced IL-1β production and release in the PBMC of celiac patients. In particular, the role of the signaling molecules underlying *de novo* synthesis of pro-IL-1β [especially the role of TLRs, MyD88 and Toll-IL-1 receptor domain-containing adaptor-inducing interferon-β (TRIF); the role of MAPK JNK, ERK and p38 MAPK; the role of NF-κB and the mechanisms of caspase-1 activation culminating in IL-1β production] were studied.

## Materials and Methods

### Abs and Reagents

Glybenclamide, KN-62, N-Acetyl-L-cysteine (NAC), quinidine and polymyxin B were from Sigma-Aldrich (St. Louis, MO, USA). Benzyloxycarbonyl-Tyr-Val-Ala-Asp-(OMe) fluoromethylketone (Z-YVAD-fmk) was from Santa Cruz Biotechnology (Santa Cruz, CA, USA). α-amylase inhibitor (AI) from *Triticum aestivum* type I and III were from Sigma. The p38 MAPK inhibitor SB203580, JNK inhibitor SP600125, serine-protease inhibitor N-p-Tosyl-L-phenyl-alanine chloromethyl ketone (TPCK) (all Sigma), and the ERK inhibitor UO126 (Cell Signaling Technology, Danvers, MA, USA) were dissolved in DMSO (Sigma). The FLICA Caspase-1 Assay kit was from ImmunoChemistry Technologies (Bloomington, MN, USA). Anti-human IL-1 β/IL-1F2 Ab and anti-mouse IL-1β/IL-1F2 Ab were from R&D Systems (Minneapolis, MN, USA), while anti-human cleaved IL-1β Ab was purchased from Cell Signaling Technology. Anti-caspase-1 (A19), anti-caspase-1 p10 (C20), and anti-beta actin were from Santa Cruz Biotechnology; anti-hTLR4-IgG(W7C11) and anti-hTLR2-IgA neutralizing Ab were from InvivoGen (San Diego, CA, USA). The following mAbs were obtained from BioLegend (San Diego, CA, USA): PE-Cy7-conjugated rat anti-mouse CD40 (3/23), BV-421-conjugated hamster anti-mouse CD80 (16-10A1), and Alexa Fluor488-conjugated rat anti-mouse CD86 (GL-1). Isotype control antibodies were from BD Biosciences (Mountain View, CA, USA) and eFluor 625NC-conjugated hamster anti-mouse CD11c (N418) was from eBioscience (San Diego, CA, USA).

### Patients

In total, 81 subjects were enrolled in this study, including 39 symptomatic untreated patients with biopsy-proven celiac disease and 42 healthy controls. Men accounted for 48.2% of the patients and 51.8% of the controls. The mean age of the patients was 34.5±8.9 years; the controls had a similar age distribution. Written informed consent was obtained from all patients and the study was approved by the Ethics Committee of the General Teaching Hospital, Prague.

### Peptic Digest of Wheat Gliadin Fraction (PDWGF)

Peptic digest of wheat gliadin fraction (gliadin, Sigma-Aldrich, St. Louis, MO, USA) and ovalbumin (Hyglos, Bernried am Starnberger See, Germany) were prepared using pepsin agarose gel (MP Biomedicals, Illkirch Cedex, France) as described previously [22]. E-toxate test for LPS (Sigma) revealed that PDWGF contained less than 2 pg/ml endotoxin.

### Isolation and Treatment of Monocytes and PBMC

PBMC and their fraction monocytes were isolated from the peripheral blood of CD patients and healthy volunteers, as described previously [23], and seeded in complete RPMI 1640 (Cambrex, East Rutherford, NJ, USA). PBMC or monocytes (2×10^5^/400 µl) were incubated for 24 h in 24-well plates (Corning, Corning, NY, USA) in the presence of PDWGF (50, 100, 250 µg/ml). All reagents were tested by the E-toxate test for LPS (Sigma) and shown to be below the limit of detection (2 pg/ml). When indicated, Z-YVAD-fmk (10 µM), quinidine (100 µM), glybenclamide (100 µM), KN-62 (10 µM), NAC (30 mM), TPCK (25 µM), SP600125 (10 µM), SB203580 (20 µM), and UO125 (10 µM) were added 30 min before gliadin stimulation. KCl (50 mM) was used to increase extracellular K+ concentration. LPS (E. coli, 0111:B4, TLR grade; Alexis Biochemicals, Farmingdale, NY, USA) was used in concentration of 100 ng/ml. In some cases, anti-hTLR4 and anti-hTLR2 neutralizing Ab (10 µg/ml, Invivogen) were added 30 min before PDWGF stimulation. In experiments using PmB, PDWGF and/or LPS was incubated with 10 µg/ml PmB for 1 h at 4°C before addition to the cells. When indicated, PDWGF and AI were incubated with dithiothreitol (DTT, Sigma) and subsequently alkylated with iodacetamide (Sigma-Aldrich) as previously described [3]. Reduced and alkylated (R/A) PDWGF and ATI were added to celiac PBMC in concentration of 100 µg/ml for 24 h. Cell culture supernatants were collected and stored at −80°C.

### Mouse BMDC Preparation

WT C57BL/6 and C57BL/10, as well as mice deficient for NLRP3, ASC, IL-1R, MyD88 and TRIF (all on a C57BL/6 background) and mice deficient for TLR2, TLR4, and TLR2/4 (all on a C57BL/10 background), were bred under specific pathogen-free conditions in the animal facilities of the Max Planck Institute of Immunobiology and Epigenetics and the University Freiburg Medical Center. All of the experimental procedures were in accordance with institutional, state and federal guidelines on animal welfare and every effort was made to minimize suffering. The animal experiments were approved by the Regierungspräsidium Freiburg and supervised by the Animal Protection Representatives of the University Freiburg Medical Center or the MPI. Mice were anesthetized before sacrificing with 1% pelltobarbitalum natricum at the dose of 10 mg/kg. BMDC were prepared from bone marrow cells obtained from the femur and tibia in RPMI 1640 (Lonza, Basel, Switzerland) and 10% FBS (Cambrex), supplemented with 20 ng/ml GM-CSF from a GM-CSF expressing line. Cells (1×10^6^ cells/ml) were washed and recultured with fresh medium containing 20 ng/ml GM-CSF every 3 d for 8 d. BMDC were cultured with PDWGF(100 µg/ml) for 21.5 h, and then 2 mM ATP (Sigma) was added for an additional 2.5 h. When indicated, Z-YVAD-fmk (10 µM) was added 30 min before PDWGF stimulation. Cells were used for flow cytometry analysis, or cell lysates were prepared and analyzed by western blot. Cell culture supernatants were analyzed by ELISA.

### ELISA

The concentrations of human IL-1β, IL-1α, IL-18 and TNF-α as well as murine IL-1β and TNF-α were measured by commercial ELISA Duo Set Kits (R&D Systems) or ELISA MAX kits (Biolegend) according to manufacturer instructions.

### FLICA Staining

Active caspase-1 was detected using the FLICA caspase-1 assay kit. Briefly, human PBMC (0.5×10^6^ cells/0.5 ml) were treated with PDWGF (100 µg/ml) for 16 h prior to treatment with fluorescein-labeled inhibitor Z-YVAD-fmk (10 µM) for 1 h at 37°C. Cells were washed three times and analyzed by flow cytometry on a FACSCalibur (BD Biosciences), and the data were analyzed using CellQuest software (BD Biosciences).

### Flow Cytometry

BMDC exposed to PDWGF, LPS, or OVA were stained with the relevant mAbs or isotype controls in a FACS bufer for 30 min on ice. In order to reduce non-specific Fc receptor-mediated binding, Fc block (CD16/CD32) from BD Biosciences was added to cells prior to and during staining. Cells were acquired on an LSR II flow cytometer (BD Biosciences) and DCs gated according to the FSC, SSC, and CD11c+ parameters for analysis.

### Western Blotting

After treatment with Z-YVAD-fmk (10 µM), quinidine (100 µM), glybenclamide (100 µM), KN-62 (10 µM), KCl (50 mM), NAC (30 mM), TPCK (25 µM), SP600125 (10 µM), SB203580 (20 µM), and UO125 (10 µM) for 30 min, cells (1×10^6^) were stimulated with PDWGF (100 µg/ml) for 24 h. Cell supernatants were collected and cell lysates were prepared as previously described [24]. Cell lysates, as well as cell supernatants, were subjected to electrophoresis on a 5–20% SDS-PAGE gradient, and then transferred to nitrocelulose membranes for western blot analysis. After blocking with 5% fat-free dried milk for 1 h at room temperature, the membranes were incubated overnight with Abs raised against pro-IL-1β, cleaved IL-1β, caspase-1, and caspase-1p10. The membranes were revealed by HRP-conjugated secondary Ab (Cell Signaling Technology, Danvers, MA, USA) using the West Femto Maximum Sensitivity Substrate (Pierce, Rockford, IL, USA). Western blot signals were detected using the LAS-1000 (Luminiscence Analyzing System; Fuji, Tokyo, Japan) and processed with AIDA 1000/1D image Analyzer software, version 3.28 (Raytest Isotopenmessgeraete, Straubenhardt, Germany). After stripping, the membranes were reprobed with anti-actin Ab (Abcam, Cambridge, USA).

### DNA Extraction

Genomic DNA was extracted from the whole blood samples of CD patients and healthy donors using a standard salting out protocol [25]. Finally, total genomic DNA was quantified with a spectrophotometer, diluted at a final concentration 20 ng/µl and stored at −20°C.

### Genotyping of NALP1 and NALP3 Polymorphisms

A single nucleotide polymorphism (SNP) in NALP1 (rs12150220) and NALP3 (rs10754558) was selected. Genotyping was performed with the 5′ nuclease assay technology for allelic discrimination using fluorogenic Taqman probes on a 7500 Fast Real Time system (Applied Biosystems, Foster City, CA, USA). Each SNP was analyzed by a specific Taqman SNP genotyping assay (Applied Biosystems). Briefly, the polymerase chain reaction (PCR) in 12 µl final volume with 20 ng of genomic DNA was performed using the ABI 7500 Thermal Cycler. The thermal cycle-sequencing conditions were as follows: initial denaturation at 95°C for 10 min, followed by 45 cycles of denaturing at 95°C for 15 s and annealing and extension at 60°C for 1 min. Data were measured and analyzed using the Applied Biosystems Software Package SDS 2.1.

### Statistical Analyses

Data were expressed as mean ± SD. Statistical analysis was performed using two-tailed Mann-Whitney U test from Graph Pad (PRISM 5.0). The statistical differences between the patient and the control groups for the genotyping of NALP1 and NALP3 polymorphism were analyzed by the Fisher’s exact two-tailed test. Relative risk was estimated by calculating the odds ratio (OR) and 95% confidence interval (95% CI). We applied a Bonferroni correction for multiple comparisons in the analysis of variant allele distributions at each SNP. A value of p<0.05 was considered to be statistically significant.

## Results

### PDWGF Induces IL-1β and IL-1α Release from Celiac Monocytes and PBMC

**Figure 1:**
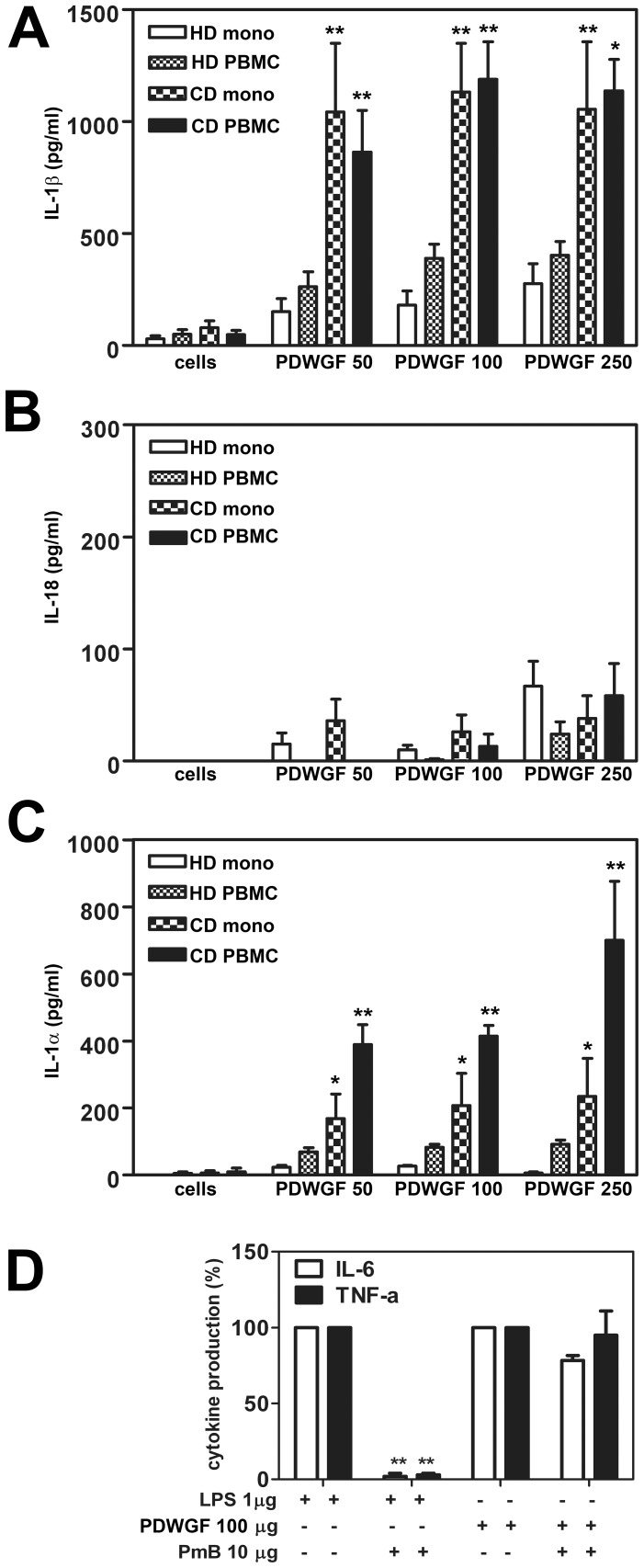
PDWGF digest induces IL-1β, IL-18, and IL-1α release in monocytes and PBMC from CD patients. IL-1β (A), IL-18 (B) and IL-1α (C) levels were quantified in cell supernatants by ELISA. Data are given as mean ± SD from 39 patients and 15 healthy donors (HD). *P<0.05, **P<0.01 (CD vs. HD). (**D**). PDWGF-induced activation of PBMC is not due to LPS contamination. Results are shown as the percentage of the cytokine production from 4 CD patients. The data were normalized to the result from untreated cells which was set as 100%. Mean ± SD, n = 4 independent experiments, ***P<0.001 compared to untreated cells.

Gliadin digest, but not α-gliadin synthetic peptides, was found to stimulate IL-1β production in celiac PBMC and its monocytes fraction [7]. To investigate the underlying mechanism, we first determined the production of IL-1β, together with other members of IL-1 family, IL-18 and IL-1α. PBMC and monocytes from active CD patients and healthy donors were exposed to PDWGF (50, 100, 250 µg/ml). Doses of PDWGF correspond to the expected concentrations of gliadin in the small intestine after a gluten-containing meal [26], [27] and were used earlier in *in vitro* studies [7], [28]. Subsequently, the secretion of IL-1β, IL-1α and IL-18 in culture supernatants was evaluated. Unstimulated monocytes and PBMC from healthy donors, as well as from celiac patients, spontaneously secreted negligible levels of IL-1β (Fig. 1A). Cells from healthy donors slightly increased IL-1β production upon PDWGF stimulation, while patientś monocytes or PBMC strongly secreted (∼2-3-fold higher) IL-1β, even already when stimulated with 50 µg/ml. In contrast to robust IL-1β production, we found measurable but far lower levels of PDWGF-induced IL-18 in cells from healthy donors and from celiac patients (Fig. 1B). Next, we tested the production of IL-1α. Celiac PBMC and monocytes secreted significantly higher levels of IL-1α, in a dose-dependent manner after PDWGF treatment, when compared to the cells from healthy donors (Fig. 1C).

To exclude that the observed effect of PDWGF is due to LPS contamination, PDWGF was preincubated with PmB for neutralization of endotoxin. LPS preincubated with PmB was used as a control. As PmB was shown to directly trigger IL-1β secretion by activating the NLRP3 inflammasome [29], we tested the effect of PmB on PDWGF-induced TNF-α and IL-6 secretion. Preincubation with PmB completely abrogated LPS-induced TNF-α and IL-6 production, but had no significant effect on PDWGF-induced TNF-α and IL-6 production by celiac PBMC (Fig. 1D).

### PDWGF Induces IL-1β Production in a Caspase-1 Dependent Manner

**Figure 2:**
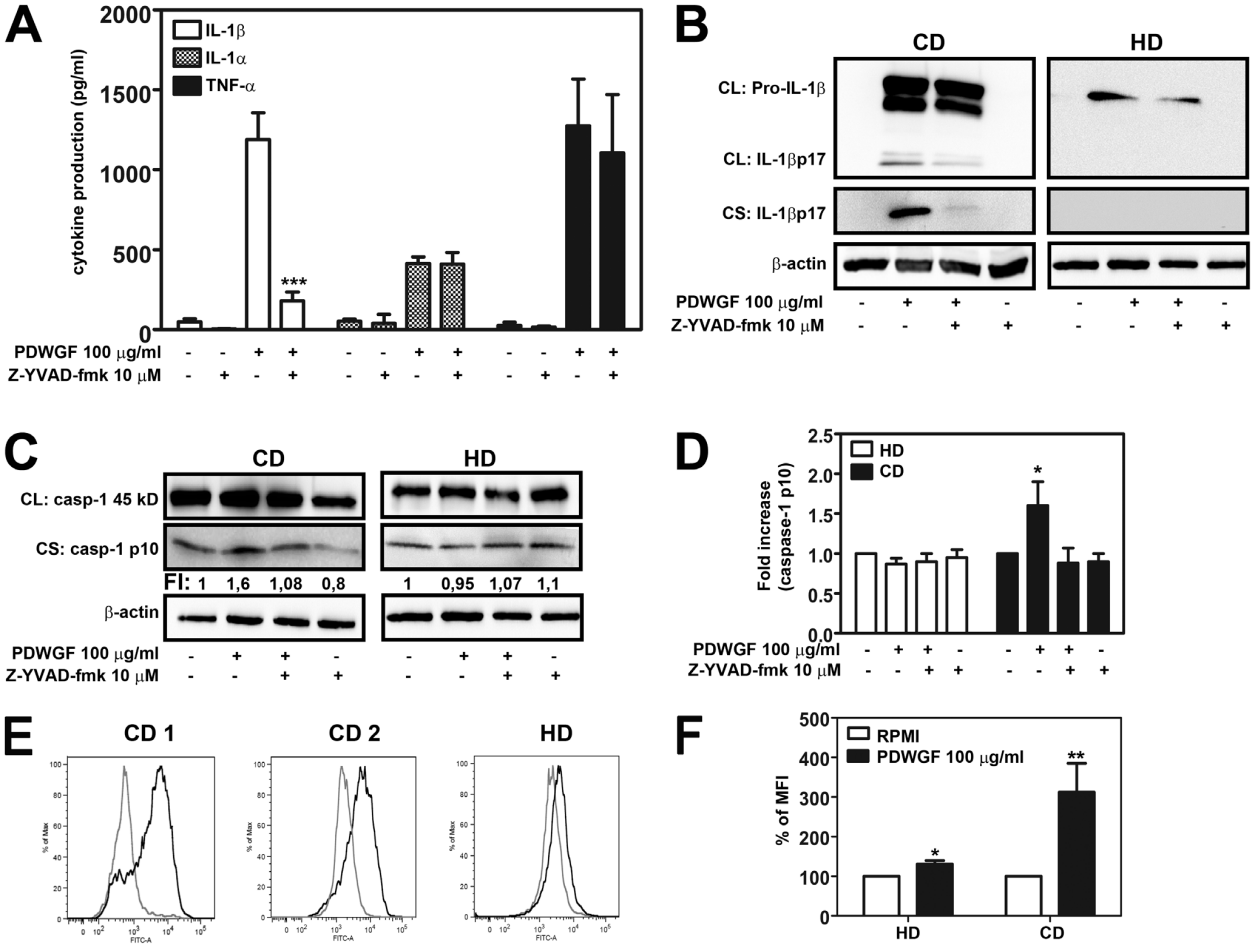
The role of caspase-1 in PDWGF-treated PBMC. (**A**) Caspase-1 inhibitor Z-YVAD-fmk reduced PDWGF-induced IL-1β, but not IL-1α or TNF-α production. Mean ± SD, n = 10 independent experiments, ***P<0.001 compared to PDWGF-treated cells. (**B**) Western blot analysis for the expression of pro-IL-1β and mature IL-1β and (**C**) for caspase-1 and caspase-1 p10 in cell lysates (CL) and cell culture supernatants (CS) from PBMC. Representative blots from 5 independent experiments are shown. β-actin was used as a loading control. (**D**) The fold increase (FI) (densitometry analysis) of the quantity of caspase-1 p10 normalized to non-activated cells. Mean ± SD, n = 5 independent experiments, *P<0.05 compared to non-activated cells. (**E, F**) Direct activation of caspase-1 in PDWGF-treated PBMC, assessed by flow cytometry using a cell-permeable fluorescent probe. Results are shown as (**E**) a representative histogram from 2 CD patients and 1 HD; and (**F**) as the percentage of the MFI from 12 CD patients and 10 HD. The data were normalized to the result from untreated cells, which was set as 100%. Mean ± SD, *P<0.05 compared to untreated cells. CD, celiac disease patients; HD, healthy donors.

To determine whether caspase-1 is required for PDWGF-induced IL-1β production, we used Z-YVAD-fmk, a specific caspase-1 inhibitor. PBMC were pretreated with Z-YVAD-fmk (10 µM) for 30 min and subsequently stimulated with PDWGF for 24 h. As shown in Fig. 2A, Z-YVAD-fmk reduced PDWGF-induced IL-1β production by 70±10% in PBMC from CD patients, but had no significant effect on PDWGF-induced IL-1α and TNF-α production.

We further confirmed that PDWGF-induced IL-1β production is caspase-1 dependent by western blot analysis. As shown in Fig. 2B, celiac PBMC treated with PDWGF for 24 h up regulated the expression of intracellular IL-1β, followed by the secretion and accumulation of mature IL-1β into supernatants. Caspase-1 inhibitor Z-YVAD-fmk strongly reduced the amount of the processed form of IL-1β in cell lysates, as well as supernatants, but had no effect on pro-IL-1β expression. In contrast, PDWGF stimulation of PBMC from healthy donors led to weak pro-IL-1β production with no detectable mature 17 kDa IL-1β form in cell lysates, nor cell culture supernatants.

We further showed that PDWGF induces processing of caspase-1 by the detection of an increased amount of the cleaved p10 subunit of caspase-1 in the conditioned supernatants from celiac, but not in healthy PBMC treated with PDWGF (Fig. 2C, D). PDWGF-induced elevated levels of caspase-1 subunit p10 were reduced in the presence of Z-YVAD-fmk. Finally, the activation of caspase-1 by PDWGF was determined by flow cytometry analysis, using a cell-permeable fluorescent probe that forms a covalent link with activated caspase-1. Our results revealed that PDWGF alone markedly increased caspase-1 activation in all celiac patients tested (mean increase of 212% ±73%, ranging from 52% to 515%). In healthy donors, the PDWGF-treated cells revealed a slight increase in caspase-1 activation (mean increase of 30% ±9%, ranging from 8% to 59%) compared to unstimulated cells (Fig. 2 E, F).

### PDWGF-induced IL-1β Secretion is Dependent on Potassium Efflux and ROS Production

**Figure 3:**
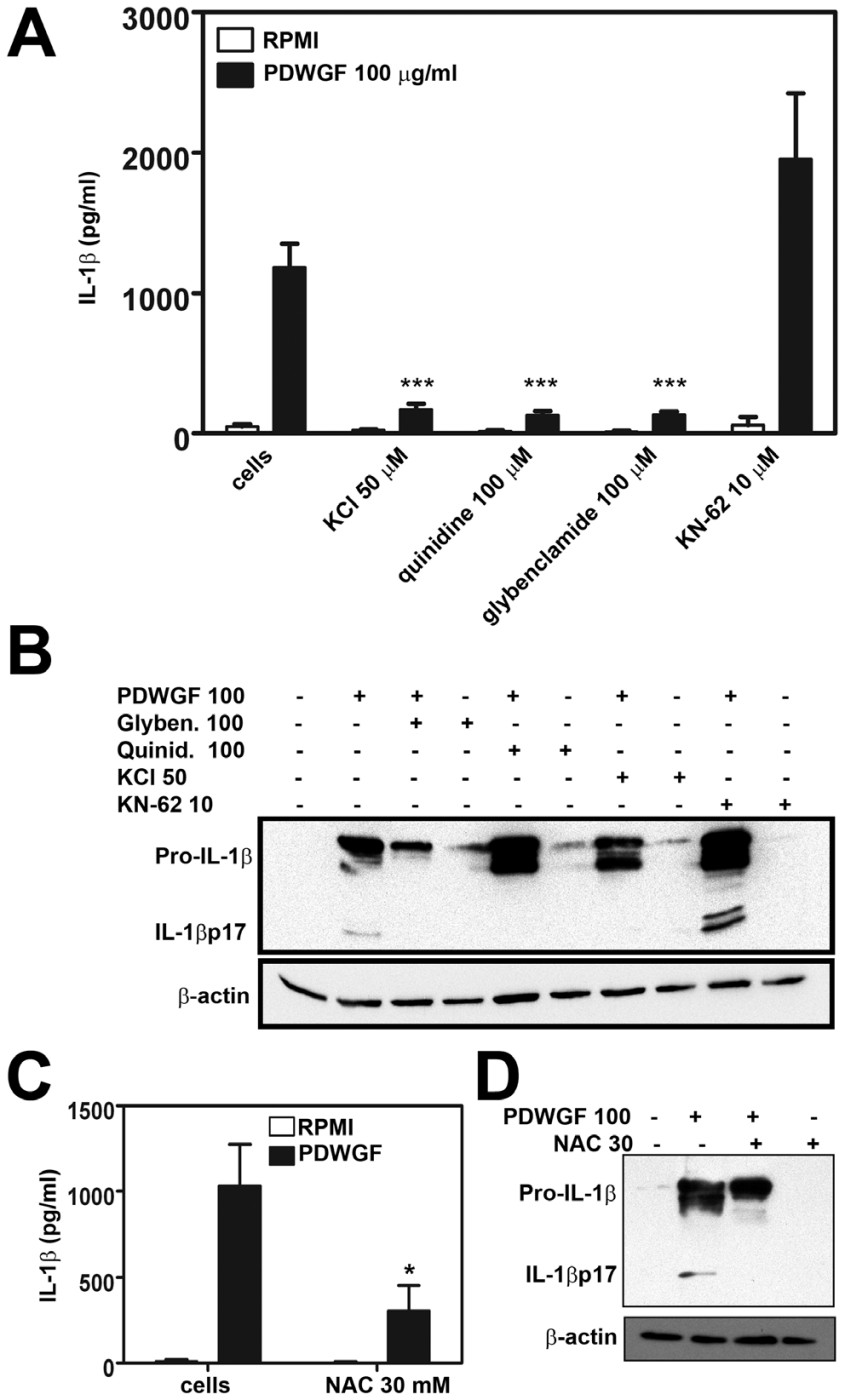
PDWGF-induced IL-1β production from celiac patient PBMC is modulated by K+ efflux, but is independent of the P2X7 receptor; as shown by (A) ELISA, mean ± SD, n = 10, ***P<0.001 vs. PDWGF-treated cells; and by (B) Western blot. Representative blots from 5 independent experiments are shown. (**C**) Inhibition of ROS modulate PDWGF-induced IL-1β secretion, mean ± SD, n = 10; as well as (D) pro-IL-1β production from PBMC of CD patients. Representative blots from 3 independent experiments are shown. β-actin was used as a loading control. ***P<0.001 vs. PDWGF-treated cells.

To test if PDWGF-induced IL-1β secretion involves the potassium efflux from cells, we exposed PBMC to a medium containing 50 mM potassium chloride, which impedes potassium efflux. Under this condition IL-1β secretion was markedly reduced upon PDWGF stimulation (Fig. 3A). To confirm the effect of potassium efflux on IL-1β induction, we treated the cells with quinidine, a potassium channel inhibitor. Thus, we demonstrated that qunidine (100 µM) significantly decreased PDWGF-induced IL-1β production (Fig. 3A). When glybenclamide (100 µM) – another inhibitor of K+ efflux – was applied, a similar effect was seen. Next, we tested if PDWGF-induced IL-1β secretion required the P2X7 receptor. We used KN-62, a potent inhibitor of ATP-induced P2X7 receptor activation. Pretreatment of cells with KN-62 (10 µM) did not lead to a decrease of PDWGF-induced IL-1β production, suggesting that PDWGF may directly trigger potassium efflux, bypassing the P2X7 receptor (Fig. 3A). Moreover, western blot analysis revealed that KCl, quinidine, and glybenclamide, but not KN-62, prevented IL-1β processing, as indicated by the failure to detect 17 kD IL-1β inside cells treated with PDWGF in combination with KCl or quinidine or glybenclamide (Fig. 3B).

Next, we tested if PDWGF might exert its effect on IL-1β production, not only by inducing K+ efflux, but also by inducing oxidative stress. PBMC were incubated for 30 min with ROS scavenger N-acetylcysteine (NAC) and stimulated with PDWGF for an additional 24 h. We found that PDWGF-induced mature IL-1β secretion detected by ELISA (Fig. 3C), as well as pro-IL-1β production detected by western blot (Fig. 3D), were markedly decreased when PBMC were stimulated with PDWGF digest combined with NAC; thus indicating that ROS may play a vital role in PDWGF-triggered IL-1β secretion (Fig. 3C, D).

### The Genotype Frequencies of ***NALP1 and NALP3*** Polymorphism Among Celiac Patients and Control Subjects

As recent studies suggested the important role of NALP1 and NALP3 genes in the predisposition to autoimmune disorders, we evaluated the possible association of single nucleotide polymorphisms (SNPs) in NLRP1 and NLRP3 genes in celiac patients and in healthy individuals. Our data (Table 1) show a significant difference in the frequencies of the SNP rs10754558 GG NALP3 genotype between the controls and patients. These data suggest that the GG genotype of NALP3 gene could play a protective role in coeliac disease (the patients group N = 1, 2.5%; the control group N = 9, 21.4%). Assessment of NALP1 and NALP3 genotype combinations in patients and control does not confirm any statistical difference between these two groups (data not shown).

**Table 1:** Genotype frequencies for *NALP1* (rs12150220) and *NALP3* (rs10754558).

	Genotype/	Control (%)	Case (%)	P-value	OR	95% CI
	Allele	n (42)	n (39)			
*rs 12150220*	AA	13 (30.9%)	10 (25.6%)	0.6252	ns	ns
	AT	19 (45.2%)	15 (38.5%)	0.5052	ns	ns
	TT	10 (23.9%)	14 (35.9%)	0.4962	ns	ns
	A	45 (40.5%)	35 (44.9%)	0.2764	1.418	0.7634–2.6333
	T	39 (46.5%)	43 (55.1%)			
*rs 10754558*	CC	17 (40.5%)	16 (41.0%)	1.0000	ns	ns
	CG	16 (38.1%)	22 (56.4%)	0.2573	ns	ns
	GG	9 (21.4%)	1 (2.5%)	**0.0141**	**0.0877**	**0.01501–0.731**
	C	50 (59.5%)	54 (69.2%)	0.2510	0.6536	0.3415–1.251
	G	34 (40.5%)	24 (30.8%)			

OR- odds ratio, CI- confidence interval.

### PDWGF Digest Induces Pro-IL-1β Synthesis via the MAPK-NF-κB Pathway

**Figure 4:**
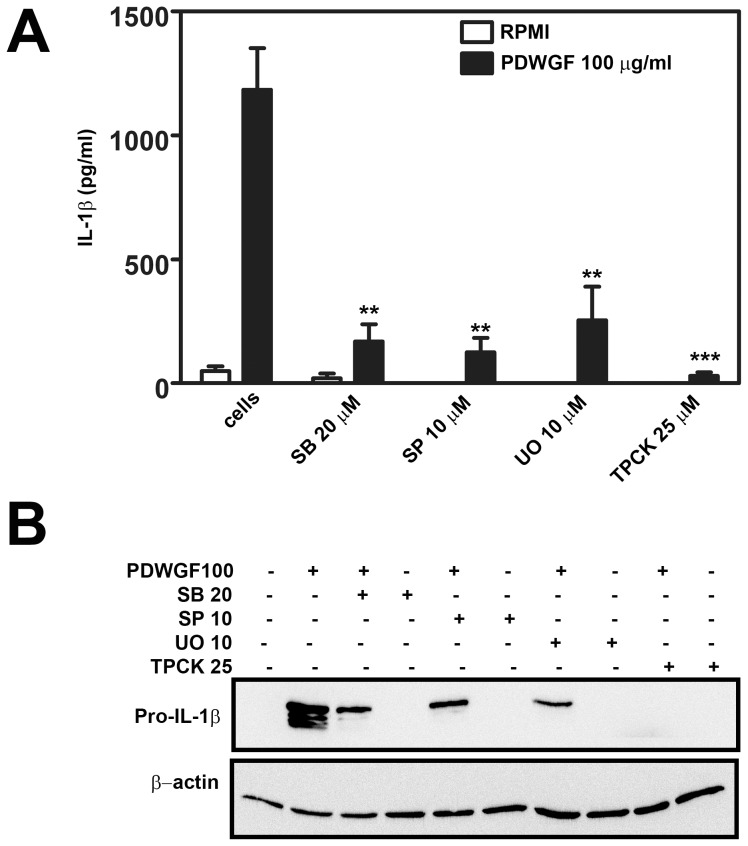
MAPK and NF-κB are involved in PDWGF mediated IL-1β secretion. PDWGF-primed PBMC were treated with SB203580 (SB), SP600125 (SP), UO125 (UO), TPCK, or PDWGF, alone or in combination for 24 h. (**A**) IL-1β was quantified in cell supernatants by ELISA. Mean ± SD, n = 5 independent experiments. ***P<0.001 vs. PDWGF-treated cells. (**B**) Pro-IL-1β levels were examined in cell lysates by immunoblotting from 5 experiments. β-actin was used as a loading control.

Next, we analyzed whether the NF-κB signaling pathway is involved in PDWGF-induced IL-1β production. Celiac PBMC were pretreated with serine protease inhibitor TPCK (25 µM) for 30 min and subsequently stimulated with PDWGF (100 µg/ml) for 24 h. Treatment with TPCK completely abolished PDWGF-induced IL-1β production (Fig. 4A), as well as all synthesis of pro-IL-1β (Fig. 4B) after PDWGF stimulation, indicating that NF-κB is a critical player during PDWGF-induced processes leading to pro-IL-1β synthesis and IL-1β release.

Furthermore, we tested if PDWGF-mediated phosphorylation of MAPKs is an upstream event leading to *de novo* synthesis of pro-IL-1β. PBMC were pre-treated with an inhibitor of p38 MAPK SB203580 (20 µM), an inhibitor of JNK SP600125 (10 µM), and an inhibitor of ERK UO125 (10 µM) for 30 min and then stimulated with PDWGF for an additional 24 h.

We found that PBMC from CD patients treated with PDWGF in combination with every single MAPK inhibitor displayed markedly reduced IL-1β secretion, when compared to cells treated with PDWGF alone (Fig. 4A). Moreover, the markedly reduced synthesis of PDWGF-induced pro-IL-1β in PBMCs treated with PDWGF combined with every single inhibitor tested, was confirmed by western blot analysis (Fig. 4B).

### PDWGF Stimulates Secretion of IL-β in Mouse BMDC in a Caspase-1 Dependent Manner and Requires NLRP3 and ASC

**Figure 5:**
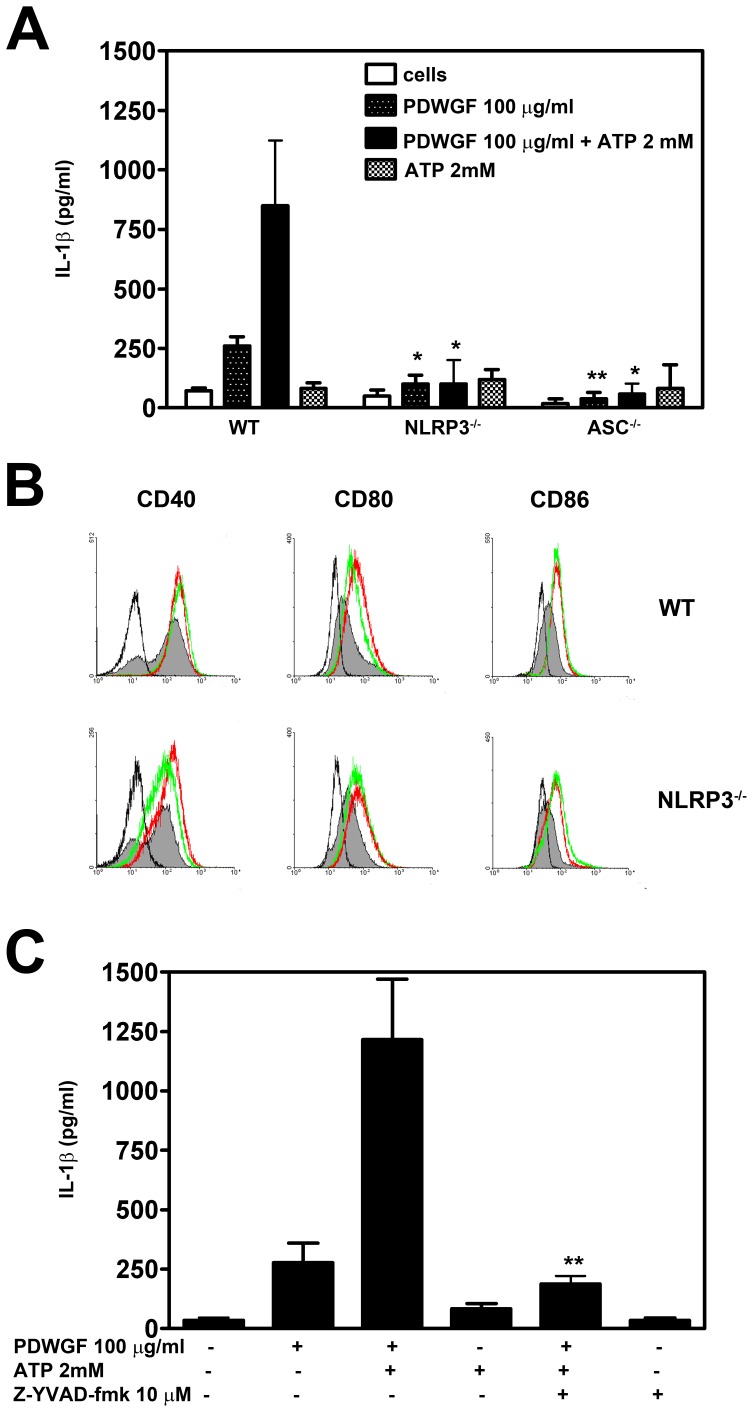
PDWGF stimulates BMDC to IL-1β production through NLRP3 and ASC. (**A**) BMDC from WT, NLRP3−/− and ASC−/− mice were exposed to PDWGF (100 µg/ml) alone for 24 h; or first PDWGF was added for 21.5 h, the subsequently ATP (2 mM) was added for additional 2.5 h. IL-1β was measured in culture supernatants. (**B**) Flow-cytometric evaluation of PDWGF-induced maturation assessed by CD40, CD80, and CD86 expression on BMDC from WT and NLRP3−/− mice. WT and NLRP3−/− BMDC were cultured with 100 µg/ml of PDWGF (green), as well as 0.1 µg/ml of LPS (red) or 100 µg/ml of OVA (grey-filled) as positive and negative controls, respectively. Isotype controls are represented in black overlays. (**C**) Cells were preincubated with caspase-1 inhibitor Z-YVAD-fmk for 30 min, and then exposed to PDWGF in combination with ATP. Production of IL-1β was measured in culture supernatants. Results are expressed as mean ± SD from 4 independent experiments. The levels of significance for KO BMDC vs. WT BMDC are indicated as follows: *P<0.05, **P<0.01, and ***P<0.001.

Our studies showing that PDWGF digest stimulates caspase-1 dependent production of IL-1β in celiac PBMC, were further extended to BMDC from mice. To analyze the role of the NLRP3 inflammasome in PDWGF-induced IL-1β release, BMDC from WT C57BL6 mice, and NLRP3−/− and ASC−/− KO mice were used. Since exogenous ATP was shown to be required for the production of mature IL-1β in macrophages and DC stimulated with TLR ligands [30], BMDC were treated with PDWGF for 21.5 h; subsequently 2 mM ATP was added for an additional 2.5 h; and IL-1β production was then determined in cell culture supernatants by ELISA. As shown in Fig. 5A, the stimulation of WT BMDC with PDWGF led to increased IL-1β secretion that was markedly elevated when exogenous ATP was added. In contrast, PDWGF and ATP-stimulated BMDC – deficient in NLRP3 or ASC proteins – secreted very low levels of IL-1β compared to the WT BMDC. On the other hand, NLRP3 and ASC molecules were dispensable for the PDWGF-induced production of TNF-α (data not shown), indicating an unimpaired cytokine production capacity of BMDC from these mouse strains. Additionally, in contrast to the induction of IL-1β, flow cytometric analysis of maturation markers (CD40, CD80, CD86) on BMDC revealed that PDWGF and LPS (as positive control) induced a similar increased maturation of BMDC, and that this effect was not dependent on the inflammasome component NLRP3 (Fig. 5B). Moreover, we found that, similar to human PBMC, treatment of mouse BMDC with caspase-1 inhibitor Z-YVAD-fmk resulted in a marked decrease of IL-1β production from PDWGF and ATP-treated cells (Fig. 5C). These data suggest that PDWGF digest induces IL-1β production through the NLRP3 inflammasome complex, and that caspase-1 is necessary for IL-1β secretion in BMDC.

### PDWGF Stimulates BMDC to IL-1β Production through TLR, MyD88 and TRIF

**Figure 6:**
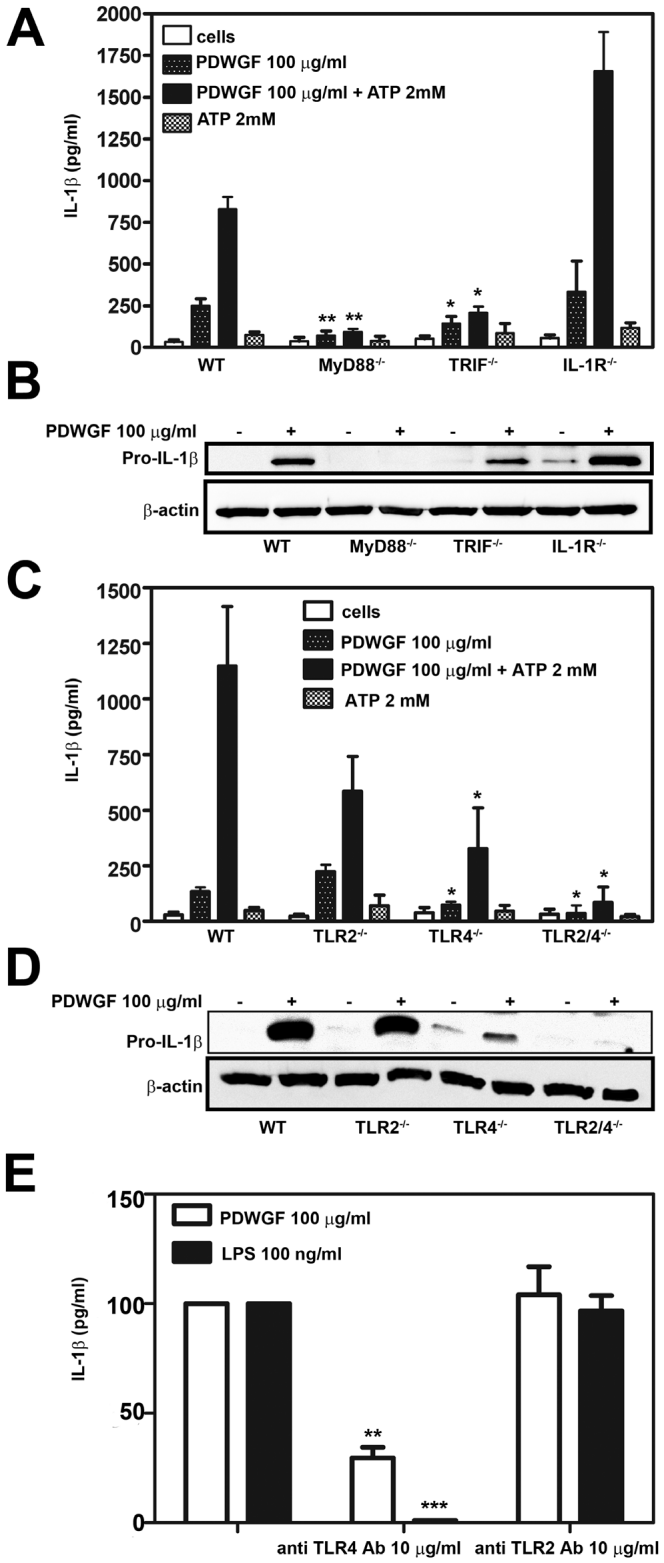
TLR signaling is required for pro-IL-1β synthesis in response to PDWGF. WT BMDC and MyD88−/−, TRIF−/−, and IL-1R−/− KO BMDC were treated with PDWGF alone or in combination with ATP and (**A**) IL-1β production was evaluated after 24 h. (**B**) Cell lysates were evaluated for *de novo* synthesis of pro-IL-1β (**C**) WT BMDC and TLR2−/−, TLR4−/−, and TLR2/4−/− KO BMDC were treated with PDWGF alone or in combination with ATP, and IL-1β production was evaluated after 24 h. (**D**) Cell lysates were evaluated for *de novo* synthesis of pro-IL-1β. Data in (A) and (C) are expressed as mean ± SD from 5 independent experiments. *P<0.05, **P<0.01 vs. WT BMDC. Blots in (B) and (D) are representative from 3 independent experiments. β-actin was used as a loading control. (**E**) Celiac PBMC were treated with PDWGF alone or in combination with anti-TLR4 or anti-TLR2 Ab. IL-1β secretion was evaluated after 24 h. LPS was used as a positive control. Mean ± SD, 8 independent experiments. **P<0.01, ***P<0.001 vs. cells without anti-TLR Ab.

Our data showing the involvement of MAPKs JNK, ERK, and p38, and of the NF-κB signaling pathway in PDWGF-induced IL-1β production from celiac PBMC (Fig. 4), led us to study upstream TLR signaling. First, we assessed the role of the MyD88 adaptor molecules, as well as TRIF, in the induction of IL-1β by PDWGF. BMDC from C57BL10 WT, MyD88, and TRIF KO mice were stimulated with PDWGF (100 µg/ml) and ATP (2 mM), as described above. We found that the secretion of IL-1β induced by PDWGF alone or in combination with ATP, was significantly reduced in MyD88 and TRIF-deficient BMDC (Fig. 6A). Consistently, the induction of pro-IL-1β in response to PDWGF was abrogated in MyD88 KO BMDC and markedly reduced in TRIF KO BMDC (Fig. 6B). In contrast, the induction and secretion of IL-1β was not reduced in BMDC deficient in IL-1R (Fig. 6B). These results suggest that TLR signaling is essential for pro-IL-1β induction in response to PDWGF digest.

By analyzing PDWGF-induced IL-1β secretion in TLR2, TLR4, and TLR2/4 KO mice, we found that the secretion of IL-1β, induced by PDWGF, alone or in combination with ATP, was significantly reduced in TLR4 KO BMDC and abrogated in BMDC from mice deficient for both TLR2 and TLR4 (Fig. 6C). Consistently, the induction of pro-IL-1β in response to PDWGF was significantly reduced in BMDC deficient in TLR4, but not TLR2 (Fig. 6D). On the other hand, BMDC from mice deficient for both TLR2 and TLR4 displayed completely abrogated pro-IL-1β production after stimulation with PDWGF, suggesting some additional role of TLR2. The findings on PDWGF-induced pro-IL-1β and IL-1β production in mouse BMDC were further confirmed in a human system, as anti-TLR4 mAb substantially reduced PDWGF-induced IL-1β release by 70% in celiac PBMC, while anti-TLR2 mAb revealed no effect on PDWGF-induced IL-1β release (Fig. 6E).

**Figure 7:**
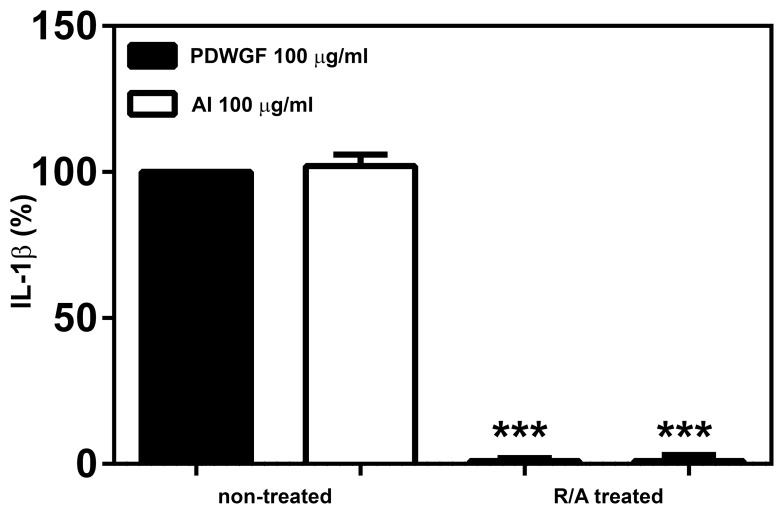
Reduction and alkylation (R/A) of PDWGF led to abrogated IL-1β production. PDWGF as well as α-amylase inhibitor (non-treated or R/A treated) were added to celiac PBMC. IL-1β secretion was evaluated after 24 h. Results are shown as the percentage of the cytokine production from 5 CD patients. The data were normalized to the result from PDWGF-treated cells which was set as 100%. Mean ± SD, 5 independent experiments. ***P<0.001 vs. non-treated PDWGF.

Very recently, Junker *et. al*. [3] reported that nongluten wheat amylase inhibitors (AI) that copurify with ω-gliadins are present in gliadin digest and can stimulate IL-8 cytokine production via TLR4 pathway. Moreover, the highly disulfide-linked secondary structure of AI is necessary to activate TLR4. Thus, we tested whether wheat AI are able to stimulate IL-1β secretion in our system. We found that AI stimulated celiac PBMC to robust secretion of IL-1β, comparable to those induced by PDWGF (Fig. 7). Next, we evaluated whether reduction and alkylation of AI as well as PDWGF will affect the capacity to induce IL-1β secretion. We found that reduction and alkylation of AI as well as PDWGF completely abolished IL-1β secretion from celiac PBMC (Fig. 7).

## Discussion

We have shown here, in line with previous study [7], that PDWGF is capable of inducing robust IL-1β production by monocytes and PBMC in celiac patients. Moreover, we show for the first time that PDWGF induces significantly increased amounts of IL-1α from monocytes and PBMC in celiac patients, and slightly elevated amounts of IL-18.

Next, we investigated the molecular mechanisms underlying the PDWGF-induced production of IL-1β in celiac PBMC. In this study we clearly document that PDWGF-induced IL-1β production by celiac PBMC is caspase-1 dependent. Interestingly, we observed that active caspase-1 was already present in unstimulated PBMC and PDWGF was able to markedly increase caspase-1 activation and the processing of pro-IL-1β in celiac PBMC, in contrast to those of healthy donors. Our data correlate with prior findings that celiac patients display the active form of caspase-1 and mature IL-18 protein in small bowel mucosa [31]. Moreover, we have confirmed that PDWGF-induced IL-1β release was dependent on NLRP3 and ASC, as shown by the stimulation of NLRP3^−/−^ and ASC^−/−^ BMDC. The role of the NLRP molecule in the predisposition to, or progression of CD remains unclear. Our data propose that the GG genotype of the SNP rs10754558 NALP3 gene could play a protective role in celiac disease. Interestingly, a similar trend has been seen in the first study of Pontillo *et al*. [32], where the G allele was protective against the development of CD (non-significantly), but this effect has not been confirmed in their following study [33]. However, similarly with our observation, neither study has found any NALP1 allele/genotype association with autoimmune disease.

Next, our study illustrated that IL-1β production from celiac PBMC is dependent on K+ efflux, since pro-IL-1β processing, as well as secretion of IL-1β were reduced when K+ efflux was impeded. In human blood monocytes, K+ efflux is mediated through membrane pores formed by P2X7 molecules after activation by autocrine released endogenous ATP [18]. Surprisingly, our data did not confirm the role of the P2X7 receptor in gliadin-induced IL-1β production in celiac PBMC, since KN-62 did not reduce either processing or secretion of gliadin-induced IL-1β. This conflict can be explained by a two step interleukin IL-1β secretion from human PBMC through both P2X7-dependent, as well as P2X7-independent mechanisms [34]. Our data show that prolonged (24 h) exposure to PDWGF, leading to elevated IL-1β secretion, does not involve the P2X7 receptor. When we tested PDWGF-induced IL-1β production after 3 h, the levels of IL-1β were below the detection limit, and we could not test the effect of KN-62. On the contrary, KN-62 markedly reduced the release of IL-1β by PBMC primed for 3 h by PDWGF and then treated with ATP for 30 min. This data suggests that the early release of PDWGF-induced IL-1β might be mediated via P2X7 receptor (data not shown).

We have further demonstrated that NF-κB is a critical player during the PDWGF-induced processes leading to pro-IL-1β synthesis and IL-1β release in celiac PBMC. Moreover, we show that in addition to NF-κB, MAPK pathways orchestrate PDWGF-induced *de novo* synthesis of pro-IL-1β in celiac cells. These data are in line with our previous studies showing that pepsin digest of gliadin triggers the activation and maturation of human DCs and PBMC via NF-κB and the p38 MAPK pathway [23], [35].

The nature of the receptors engaged by gliadin digests as well as gliadin peptides (or other wheat components) responsible for mediating innate immunity remains to be clearly identified. Gliadin is a complex protein containing multiple epitopes that exert cytotoxic, immunomodulatory and intestinal permeability effects. Some gluten-derived peptides elicit T-cell specific responses, while some other “non-immunodominant” gliadin derived peptides stimulate innate immunity response in the gut [36]–[39]. The chemokine receptor CXCR3 was shown to serve as a receptor for specific gliadin peptides that cause zonulin release and subsequent increase in intestinal permeability [40]. Next, MyD88 was shown to be involved in gliadin-induced inflammatory cytokine production in mouse macrophages [6]. In our study, PDWGF-induced IL-1β production was strongly dependent on MyD88, and to certain extend on TRIF, which suggests that components of gliadin digest could induce signaling via TLR receptors. As LPS contamination was ruled out in our study, it seems that the stimulatory effect of our gliadin preparation is caused by proteins. In mouse models, TLR2 and TLR4 were not identified as a component of gluten or gliadin-induced signaling pathway [6], [41]. On the other hand, very recently, Junker *et al*., [3] reported that activation of innate immune responses by pepsin trypsin gliadin digest is due to wheat amylase trypsin inhibitors CM3 and 0.19 that signal via TLR4/CD14 complex. Moreover, the highly-disulfide linked secondary structure of amylase trypsin inhibitors is very important for activation of TLR4. Here, we documented that reduction of our PDWGF led to completely abrogated capacity to stimulate IL-1β production in PBMC. It suggests that amylase trypsin inhibitors may be present in our PDWGF and they may orchestrate in IL-1β production. We also documented that BMDC from TLR2−/− mice produced slightly reduced IL-1β levels, whereas BMDC from TLR4−/− mice displayed significantly decreased IL-1β levels after the PDWGF triggering. Interestingly, cells deficient for both TLR2 and TLR4 displayed completely abrogated IL-1β production, indicating that the combined action of TLR2 and TLR4 may be involved in PDWGF signaling. Notably, the combined action of the two TLRs was shown to be required for sensitization to contact allergens in a murine model of allergic contact dermatitis [42], and for the regulation and tissue repair in bleomycin-induced mouse tissue injury [43]. Our murine data, suggesting a role of TLR in the signaling pathway underlying IL-1β production, was confirmed in humans using anti-TLR4 Ab that markedly reduced PDWGF-induced IL-1β in celiac PBMC. The possible role of TLR involvement is supported by the fact that celiac patients were found to have higher expression of TLR2 and TLR4 molecules in intestinal tissue [44]. Moreover, they exhibited a higher prevalence of TLR2 and TLR4 positive blood DCs and monocytes compared to controls [45]. In contrast, recently other group [46] reported decreased levels of TLR receptors in the small intestinal biopsies of celiac patients.

However, as we did not observe a complete inhibition of TLR signaling using the anti-TLR4 antibody in PBMC, it is plausible that also here TLR2 and/or other signaling molecules may be involved in the activation of human cells in response to PDWGF.

In conclusion, in accordance to recently published study [3], our findings suggest that PDWGF does not only contain T cell epitopes, but also other wheat components that could play a role as adjuvants activating the innate immune system via TLR2/4, ROS, and the NLRP3 inflammasome. In addition, T cell and NK cell responses through IFN-γ were shown to strongly potentiate the pro-inflammatory activity of TLR ligands [47]. Both mechanisms may be involved in the pathogenesis of wheat allergy as well as CD.
